# Stabilization of the classical phenotype upon integration of pancreatic cancer cells into the duodenal epithelium

**DOI:** 10.1016/j.neo.2021.11.007

**Published:** 2021-11-16

**Authors:** Benedek Bozóky, Fernández Moro, Carina Strell, Natalie Geyer, Rainer L. Heuchel, J. Matthias Löhr, Ingemar Ernberg, Laszlo Szekely, Marco Gerling, Béla Bozóky

**Affiliations:** a Theme Cancer, Karolinska University Hospital, Solna 17176, Sweden; b Department of Microbiology, Tumor, and Cell Biology, Karolinska Institutet, Solnavägen 9, Solna 17165, Sweden; c Department of Clinical Pathology and Cancer Diagnostics, Karolinska University Hospital, Huddinge 14186, Sweden; d Department of Laboratory Medicine, Division of Pathology, Karolinska Institutet, Huddinge 14186, Sweden; e Department of Oncology-Pathology, Karolinska Institutet, Solna 17164, Sweden; f Department of Immunology, Genetics and Pathology, Uppsala University, Uppsala 75185, Sweden; g Department of Biosciences and Nutrition, Karolinska Institutet, Hälsovägen 7, Huddinge 14183, Sweden; h Department of Clinical Science, Intervention and Technology (CLINTEC), Karolinska Institutet, Huddinge 14186, Sweden

**Keywords:** Pancreatic cancer, Transcriptome subtypes, Tumor microenvironment, Local invasion, Intestinal mimicry

## Abstract

**Introduction:**

Pancreatic ductal adenocarcinoma (PDAC) is one of the most aggressive solid tumors. Based on transcriptomic classifiers, basal-like and classical PDAC subtypes have been defined that differ in prognosis. Cells of both subtypes can coexist in individual tumors; however, the contribution of either clonal heterogeneity or microenvironmental cues to subtype heterogeneity is unclear. Here, we report the spatial tumor phenotype dynamics in a cohort of patients in whom PDAC infiltrated the duodenal wall, and identify the duodenal epithelium as a distinct PDAC microniche.

**Materials and methods:**

We used serial multiplex quantitative immunohistochemistry (smq-IHC) for 24 proteins to phenotypically chart PDAC tumor cells in patients whose tumors infiltrated the duodenal epithelium. Additionally, we used a genetically engineered mouse model to study the PDAC cell phenotype in the small intestinal epithelium in a controlled genetic background.

**Result:**

We show that pancreatic cancer cells revert to non-destructive growth upon integration into the duodenal epithelium, where they adopt traits of intestinal cell differentiation, associated with phenotypical stabilization of the classical subtype. The integrated tumor cells replace epithelial cells in an adenoma-like manner, as opposed to invasive growth in the submucosa. Finally, we show that this phenomenon is shared between species, by confirming duodenal integration and phenotypic switching in a genetic PDAC mouse model.

**Discussion:**

Our results identify the duodenal epithelium as a distinct PDAC microniche and tightly link microenvironmental cue to cancer transcriptional subtypes. The phenomenon of “intestinal mimicry” provides a unique opportunity for the systematic investigation of microenvironmental influences on pancreatic cancer plasticity.

## Highlights

• PDAC cells in the duodenal epithelium mimic intestinal cells and co-opt the basement membrane.
• Intramucosal PDAC location is strongly coupled to the classical phenotype and to intestinal traits.
• Intratumoral heterogeneity is linked to specific tissue compartments, which shape phenotype plasticity of PDAC cells.

## Introduction

PDAC is one of the most lethal tumors with a five-year survival rate of less than 9% [1]. Although clinically perceived as uniformly aggressive, PDAC subtypes with varying clinical outcomes can be defined based on transcriptional profiling 2, 3, 4, 5, 6. Classifications distinguish two major subtypes, “classical” and “basal-like”, although intermediate states and less common subtypes exist [2]. Basal-like tumors are associated with a worse prognosis [2, 3, 4,6]. While subtyping based on bulk transcriptomic data is prognostically valuable, cells with both a basal-like and a classical phenotype co-exist in individual tumors, as revealed by single-cell RNA sequencing [2]. Genetic changes, such as allelic imbalances of mutant *KRAS*, contribute to this intratumor heterogeneity [2,7]; however, mouse models and organoid co-cultures have demonstrated a central role for the microenvironment in shaping the PDAC tumor cell phenotype [8,9], and there is strong support from experimental studies that the non-malignant microenvironment can reprogram malignant cells to a normal-like behavior [10,11]. In human PDAC, cancer cell states have recently been linked to adjacent fibroblast subtypes [12]. Nevertheless, the full extent to which microenvironmental cues shape tumor cell phenotypes remains unclear.

Routine pathological assessment regularly reveals morphological heterogeneity in pancreatic tumors [13]. In clinical cases where a small intestinal mass is biopsied endoscopically, PDAC can occasionally be misdiagnosed as an intestinal neoplasm, or even be mistaken for reactive small intestinal changes. This rare, but clinically important phenomenon – that poses diagnostic difficulties – has previously been termed “intestinal mimicry”, and an immunohistochemical marker panel has been proposed to improve PDAC diagnosis based on intestinal biopsies [14,15].

The ability of PDAC cells to escape the pathologist's eye once settled in the duodenal epithelium suggests strong phenotypical changes in the tumor cells, while leaving the underlying stroma relatively intact. This is remarkable, given the destructive mode of growth and the strong desmoplastic stromal reaction that otherwise characterize PDAC [13].

Based on the emerging PDAC subtypes, we have studied in detail the changes relating to intestinal mimicry that occur in the tumor cell phenotype upon switching location from the pancreas to the small intestine. We have systematically mapped tumor cell phenotype dynamics in a unique cohort of PDAC patients with duodenal infiltration, collected over more than a decade of routine pathological diagnostics at a large tertiary care center specializing in pancreatic resections. We found that PDAC cells in the small intestinal epithelium revert to non-destructive growth, and switch to a purely classical phenotype upon epithelial integration. In the duodenal epithelium, protein expression of PDAC cells mimics that of small intestinal enterocytes, while the stroma retains its small intestinal identity devoid of desmoplasia. For the first time, our results link the small intestinal microenvironment to defined shifts in PDAC tumor cell subtypes. Together, they suggest that intestinal mimicry provides the remarkable – yet largely overlooked – possibility of studying cancer cell differentiation towards a less aggressive, near-normal phenotype in relation to microenvironmental cues and within spatially defined tissue compartments.

## Material and methods

### Patients

Patients with duodenal infiltration of primary PDAC were identified by a retrospective search in the pathology archive database of the Karolinska University Hospital, Huddinge, Sweden, and through routine diagnostic pathology between 2008 and 2020. In all cases, the tumor epicenter was located in the pancreas and all tumors were classified as PDAC histologically and by the local multidisciplinary tumor board.

Cases in which infiltration of PDAC cells into the small intestine was described in the pathology report, or for which the participating pathologists (CFM, Béla B, LS) noted this phenomenon, were selected and systematically reassessed based on available hematoxylin and eosin (H&E) staining and immunohistochemistry (IHC). Clinical data were obtained by retrospective chart review.

## Serial multiplex quantitative immunohistochemistry

Serial multiplex quantitative immunohistochemistry (smq-IHC) was performed as described previously [16]. Briefly, formalin-fixed paraffin-embedded (FFPE) samples were cut to a thickness of 4 µm and stained on an automated stainer (BOND-MAX, Leica Biosystems, Germany) as part of the diagnostic routine in a clinically accredited histology lab. Staining procedures have been described previously [16]. Antibodies and staining protocols are presented in **Supplementary Table 1**.

For quantification, H&E stains as well as IHC for Mothers against decapentaplegic homolog 4 (SMAD4) and Tumor protein 53 (p53) guided the identification of tumor regions and individual cells, which were then manually matched to corresponding regions in serial sections, while SMAD4/p53 stains were used to navigate through the sections (**Supplementary Fig. 1**). All identifiable tumor cells in the mucosa and submucosa of one representative paraffin block were included in the quantification process. The average areas on which the calculations are based were: 15.3 mm^2^ (range 3.7–36.6 mm^2^) for the mucosa and 37.2 mm^2^ (range 7.0–153.9 mm^2^) for the submucosa, respectively.

Quantification was achieved by calculating the percentage of positive cells with respect to all tumor cells in the two separate regions, i.e. mucosa and submucosa.

For all antibody stains, at least *n* = 10 cases were included for the final assessment, based on staining quality and availability. For all quantified antibody stains, statistics are based on the evaluation of at least *n* = 15 matched mucosa/submucosa pairs.

This study was approved by the responsible Ethical Review Board (no. 2020-06115, *Etikprövningsmyndigheten* and 2015/259-31/2, *Etikprövningsnämnd*, Sweden).

## Mice

FFPE tissue sections from a cohort of *Kras^LSL−G12D/+^;Trp53^LSL−R172H;+^;Pdx1-Cre* (KPC) mice [17] described previously [18] were analyzed for the presence of duodenal invasion based on available H&E sections. One animal was identified where PDAC cells had infiltrated the duodenum; FFPE sections from this mouse were assessed for expression of the high-motility group AT-hook 2 (HMGA2) protein by immunohistochemistry, as described previously [18]. The antibody used is listed in **Supplementary Table 1**. Animal experiments were approved by the Swedish Board of Agriculture, Sweden, Nr. S31/15 (*Stockholms Södra Djurförsöksetiska Nämnd*).

## Results

### Consistent morphological changes in PDAC cells in the duodenum

A total of *n* = 20 patients in whom PDAC cells had infiltrated the entire thickness of the duodenal wall were identified. All patients (*n =* 8 females, *n =* 12 males, aged 64–82 years, median age 71.8 years) underwent resection according to Whipple at Karolinska University Hospital, Stockholm, Sweden. A total of *n =* 19 patients passed away during the observation time, median overall survival was *n =* 559 days after operation (range *n =* 3 to *n =* 2460 days). One patient was alive when data collection was completed in January 2021.

**Fig. 1:**
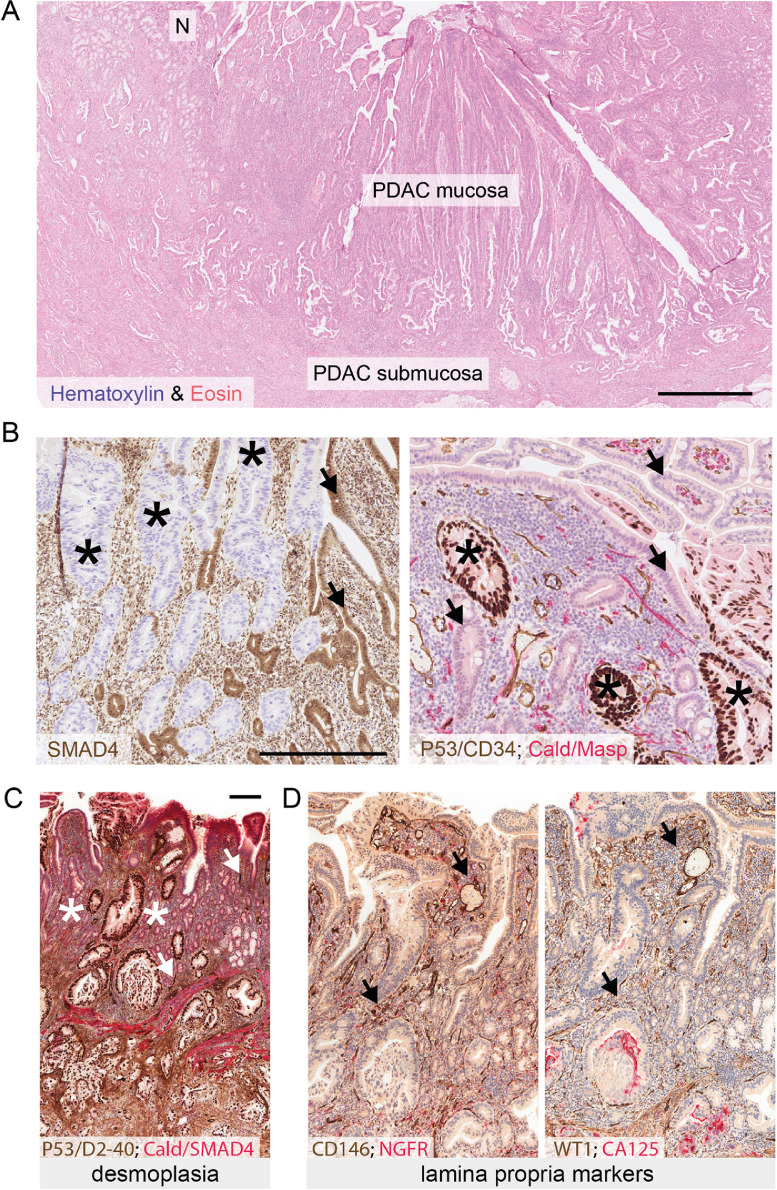
*Histology and tumor cell identification in duodenal invasion of pancreatic ductal adenocarcinoma (PDAC).***(A)** Hematoxylin & eosin (H&E) staining of PDAC cells that have invaded and integrated into the duodenal mucosa. “N” indicates non-neoplastic duodenal epithelium. Scale bar: 1 mm. (**B)** Left panel: Representative immunohistochemistry (IHC) for SMAD4 in a PDAC case with genetic loss of *SMAD4* shows intestinal villi lined by SMAD4-negative PDAC cells (asterisks) compared to adjacent small intestinal epithelial cells positive for SMAD4 expression (arrows). Right panel: Representative image of p53 IHC in a PDAC case with accumulation of p53 protein due to *TP53* mutation; note regions of p53-positive PDAC cells (asterisks) adjacent to p53-negative intestinal epithelial cells (arrows). Scale bar: 200 µm for both panels**. (C)** Immunohistochemistry (IHC) for the indicated proteins illustrating overexpression of the desmoplasia marker D2-40 (podoplanin) in the submucosa compared to the mucosa; note that in this quadruple staining, the brown stain identifies both D2-40 (stromal) and p53 (epithelial) expression, and red identifies both caldesmon (Cald, stromal) and SMAD4 (epithelial) expression; asterisks indicate stroma. Note that the rare D2-40 positive structures visible in the mucosa represent lymphatic endothelial cells (dark brown, examples indicated with arrows). (**D)** IHC for the lamina propria marker, CD146 (left panel), shows preserved expression in areas of intraepithelial tumor integration (arrows). IHC for another lamina propria marker, WT1 (right panel), also shows preserved expression in areas of tumor cell epithelial integration (arrows). Representative stainings of *n* ≥ 10 cases. Scale bar: 200 µm (applies to C and D). All IHC counterstained with hematoxylin. Multiplex staining combinations are indicated in panels, text color denotes color of chromogen used to visualize protein expression (For interpretation of the references to color in this figure legend, the reader is referred to the web version of this article.).

In patients where PDAC cells had infiltrated the duodenal epithelium, we observed consistent morphological changes that accompanied tumor cell integration into the epithelial layer (Fig. 1**A**). In all cases, the mucosal architecture was strikingly preserved, and the epithelial lining exhibited either normal morphology or mild reactive atypia, interspersed with areas of columnar epithelium with a dysplastic/neoplastic appearance, in line with the previously recognized diagnostic challenge [15]. To confirm unequivocally that the neoplastic cells in the duodenal mucosa were of pancreatic origin, rather than reactive intestinal cells secondary to PDAC infiltration into the submucosa, we used two independent immunohistochemical markers, SMAD4 and p53; SMAD4 is lost in approximately half of all pancreatic cancer cases [19]; while *TP53* mutations, which lead to accumulation of mutant p53 protein, are detectable in 50% of patients, independently of alterations in *SMAD4*^19^. Both markers confirmed the seamless integration of PDAC cells into the SMAD4^+ve^/p53^−ve/low^ duodenal epithelium, without destruction of the mucosal architecture (Fig. 1**B**).

Intramucosal PDAC cells were well differentiated and polarized, in contrast to the submucosa, which harbored pleomorphic cancer cells and irregular glands, consistent with primary PDAC histomorphology. A hallmark of PDAC is its desmoplastic stroma, in which tumor glands are embedded [12]. Notably, IHC for the desmoplasia marker, podoplanin (D2-40) [20], revealed no desmoplasia of the subepithelial stroma adjacent to mucosal cancer cell integration, while desmoplasia was present in the submucosa (Fig. 1**C**). In contrast, protein expression of CD146 and WT1 (Fig. 1**D**), markers of the intestinal *lamina propria* [21,22], was preserved in regions adjacent to intraepithelial tumor cells.

### Stabilization of the classical phenotype upon epithelial integration

The diverging phenotypes of PDAC cells in mucosal *vs*. submucosal locations implied a high degree of location-dependency, suggesting that the local microenvironment is a major contributor to the tumor cell phenotype. To quantify these phenotypic differences, we used smq-IHC to assess a panel of 12 protein markers, selected to identify tumor cell characteristics, and to approximate the transcriptional subtypes [2] (see also **Supplementary Table 2**), as previous studies had shown that condensed marker panels can differentiate between basal-like and classical subtypes with high accuracy [23]. The panel comprised intestinal as well as pancreatobiliary differentiation markers (CK20, CDX2, MUC2, MUC1, MUC5AC), all of which are included in the classical transcriptional profile [2], a pancreatobiliary marker specific to the basal-like profile (CK17), general markers for PDAC cells (CK7, MUC6) and tumor-specific glycoproteins (CA19-9, CA125, and CEA), together with Ki67 to assess proliferation.

**Fig. 2:**
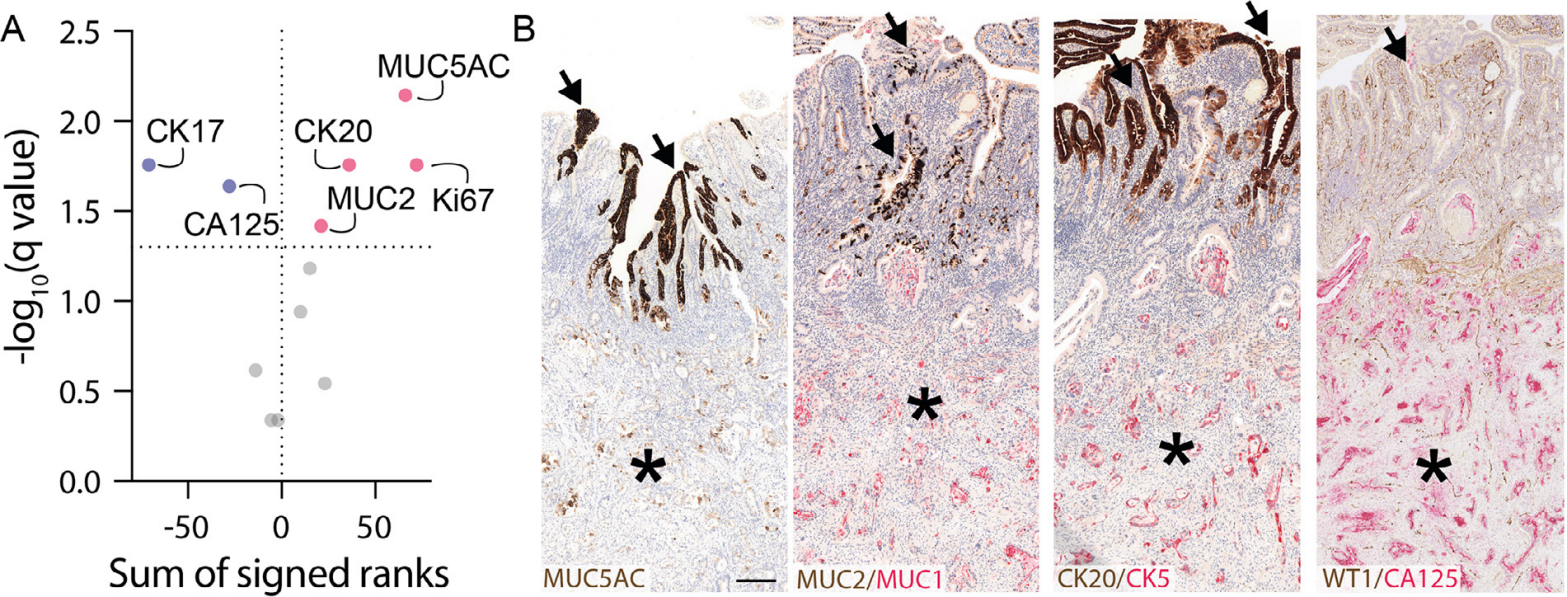
*Phenotypic shift of pancreatic cancer cells upon integration into the duodenal mucosa.***(A)** Volcano plot showing significant findings in red (higher in mucosa) and blue (higher in submucosa) out of a total of *n =* 12 markers included in the analysis. Data based on results from Wilcoxon matched-pairs signed rank test, multiple test correction with two-stage step-up method (Benjamini, Hochberg, Yekutieli) FDR < 0.05. (**B)** Differential protein expression in mucosal vs. submucosal tumor cells for MUC5AC, MUC2/MUC1, CK20/CK5, and WT1/CA125. Rightmost three images from the same patient case, for which MUC5AC was not available (leftmost patient). Scale bar: 200 µm, applies to all images in (B); representative staining of *n* ≥ 15 cases. Note that multiplex immunohistochemistry was performed for some markers, but not all markers were included in quantitative analysis (e.g. WT1). Asterisks indicate submucosa, arrows indicate areas of tumor cells that have integrated into the epithelium (For interpretation of the references to color in this figure legend, the reader is referred to the web version of this article.).

The smq-IHC results revealed significantly reduced expression of the basal-like marker, CK17, along with CA125, in the mucosa *vs.* submucosa. In contrast, the expression of the classical/intestinal markers, MUC5AC, CK20, and MUC2 was significantly increased in the mucosa compared to the submucosa (Fig. 2 **A & B, Supplementary Table 2**), supporting a strong phenotypic switch away from basal-like towards classical differentiation following intramucosal integration, which was accompanied by enhanced Ki67 positivity (Fig. 2**A**).

### Murine PDAC recapitulates phenotypic plasticity

**Fig. 3:**
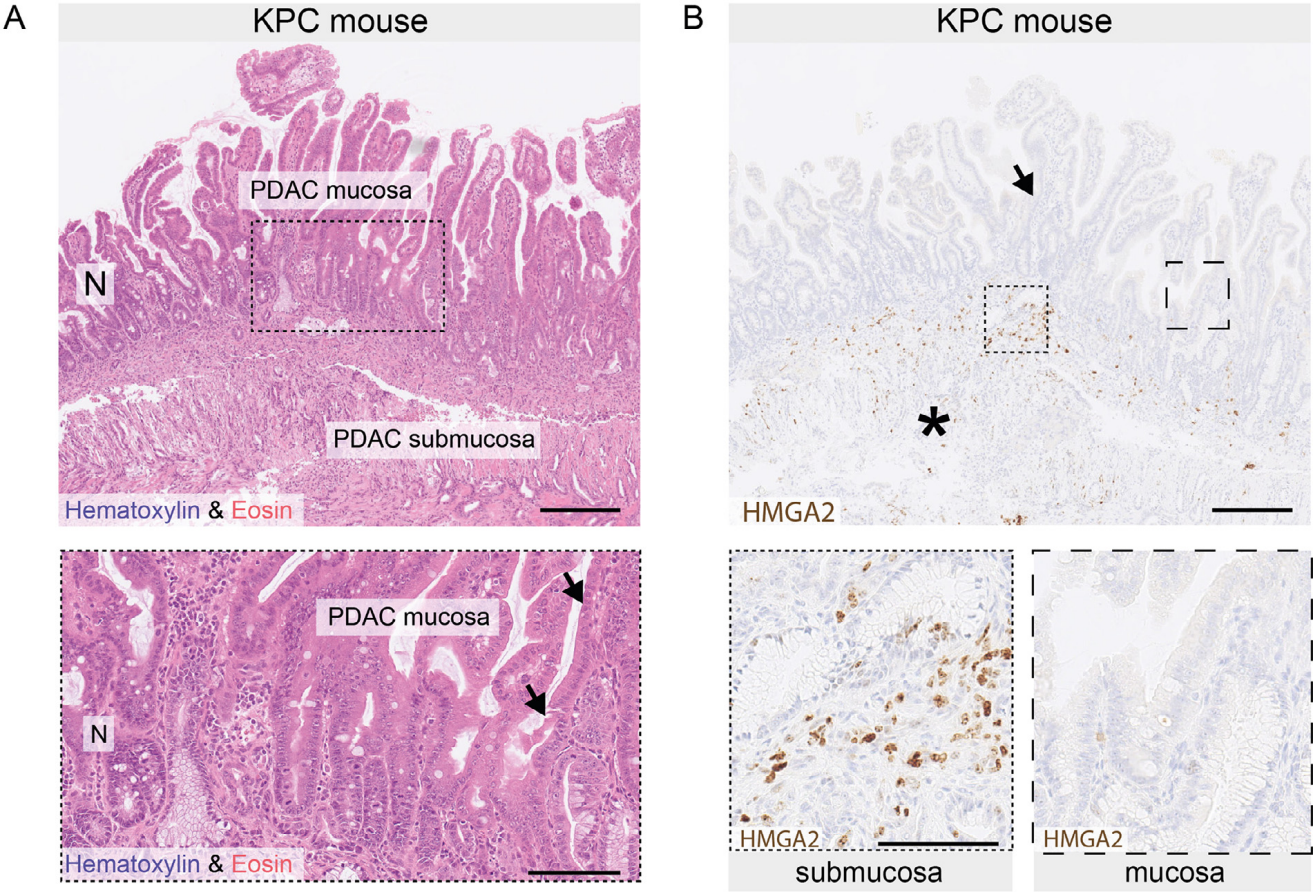
*Intestinal mimicry in a genetic mouse model of pancreatic cancer.***(A)** Hematoxylin & eosin staining of murine small intestine infiltrated by tumor cells driven by mutations in *KRAS* and *P53* (*Kras^LSL−G12D/+^;Trp53^LSL−R172H;+^;Pdx1-Cre* mice, KPC). “*N*” indicates a region of normal small intestinal epithelium, “PDAC mucosa” indicates areas where tumor cells have integrated into the epithelial layer of the intestine; tumor cells are identified based on morphology, indicated by arrows in magnified lower panel. (**B)** Immunohistochemistry for high mobility group AT-hook 2 (HMGA2) protein. HMGA2 is lost in areas of intestinal epithelial infiltration, while it is expressed in the submucosa; asterisk and arrow indicate extramucosal invasion and mucosal integration of PDAC cells, respectively. Scale bars: 250 µm for upper panels of *A* and *B*, 100 µm for lower panels of *A* and *B*.

The KPC mouse model [17] closely recapitulates PDAC morphology, driven by mutations in *Kras* and *Tp53* under the control of a pancreas-specific promoter (*Pdx1*). To assess whether small intestinal infiltration of PDAC leads to morphological changes similar to those in humans, we analyzed tissues from *n =* 6 KPC mice with PDAC. We identified one animal, in which PDAC cells infiltrated the small intestine to the level of the epithelium. Morphological changes were similar to those observed in humans, such that the tumor cells in the epithelial layer were polarized and morphologically mimicked enterocytes (Fig. 3**A**). Next, we assessed the expression of HMGA2, a transcriptional marker for basal-like tumor cells in human PDAC [2], that is also expressed in murine KPC tumors and for which staining of murine tissue has been established previously [18]. In concordance with human PDAC, HMGA2 was downregulated in murine PDAC cells located in the epithelial layer (Fig. 3**B**) compared to PDAC cells in the submucosa, consistent with the attenuation of the basal-like phenotype upon intestinal integration.

## Discussion

**Fig. 4:**
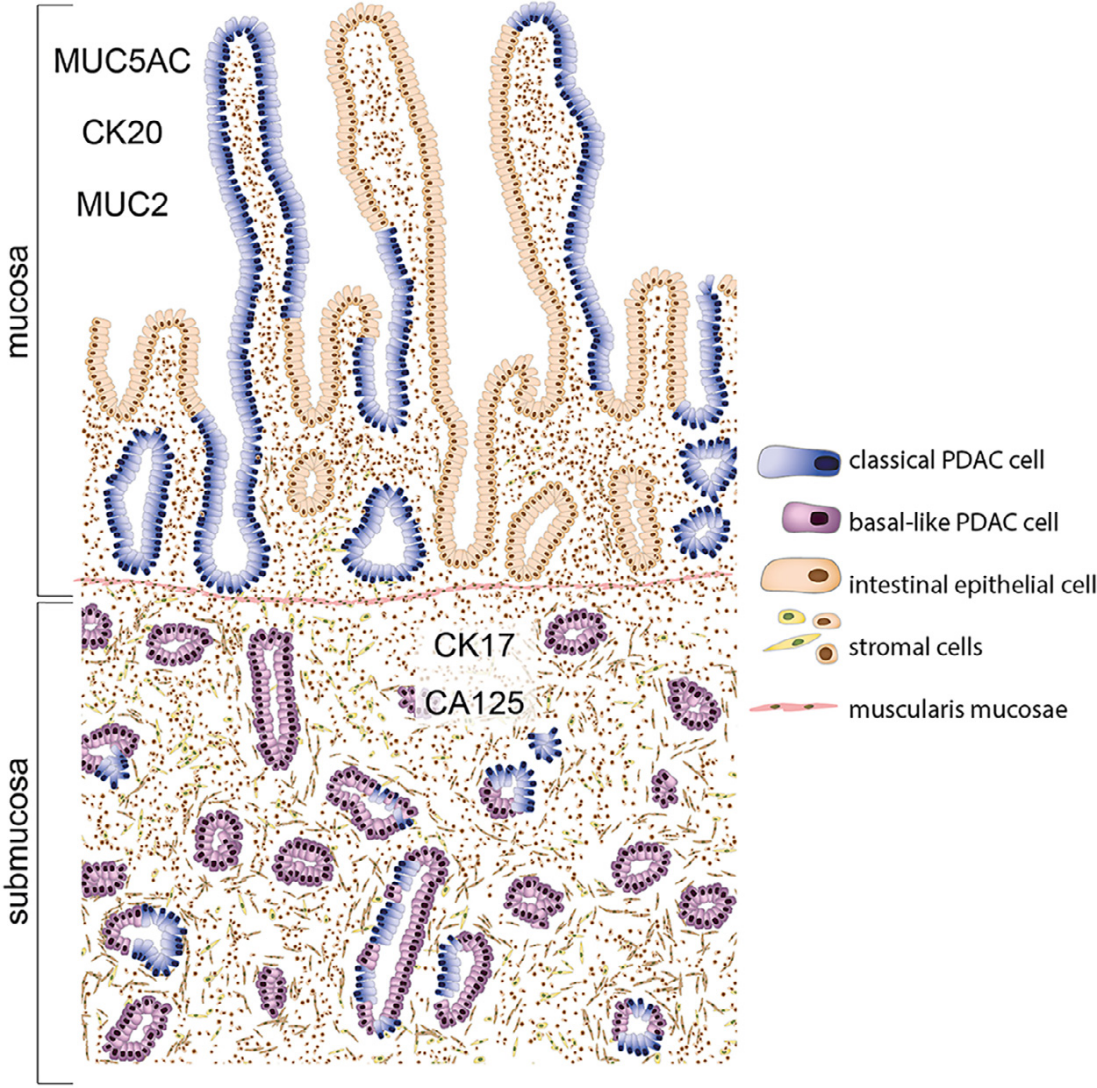
*Illustration of the phenotypic switch of pancreatic cancer cells in the duodenum.* Both basal-like and classical phenotypes co-exist in the submucosa. Cancer cells that integrate into the duodenal epithelium switch to a classical phenotype, recapitulate intestinal epithelial cell characteristics, respect the basement membrane, and grow in an adenoma-like manner.

The tumor microenvironment influences cancer development and progression, exerting both cancer-promoting and restraining effects. The extent to which tumor location – and hence the spatial relationship of tumor cells to their specific microenvironment – shapes the PDAC cell phenotype is unclear. Here, we show that the integration of PDAC cells into the duodenal mucosa is associated with a quantifiable phenotypic shift towards intestinal differentiation, identifying the duodenal epithelium as a specific PDAC microniche (Fig. 4). Location-dependent morphological changes are accompanied by a loss of basal-like subtype markers in favor of classical subtype markers, corresponding to a switch towards a less aggressive molecular phenotype, and strong intestinal cell-like differentiation of the integrated tumor cells [2, 3, 4,6]. Interestingly, mucosal PDAC cells were cycling (Ki67^+ve^) at higher levels than submucosal tumor cells, suggesting the uncoupling of differentiation from proliferation. While the increase in Ki67 positivity in conjunction with a less aggressive phenotype may appear counterintuitive, results from studies on the prognostic value of Ki67 expression in PDAC have been mixed [24,25]. The intestinal mucosa is a highly proliferative tissue that renews every five days [26] and hence, the increase in proliferative activity may be interpreted as part of the alignment of PDAC cells with their epithelial location.

Our results establish that PDAC cells integrate into the epithelial compartment of the duodenum, where they progress in an *in situ*-like manner. Importantly, for the first time, we provide evidence that this phenomenon is connected to the distinct tumor subtypes. Non-destructive growth in the epithelium requires PDAC cells to respect the native basement membrane, and it does not induce stromal desmoplasia; hence, it differs significantly from tumor growth in the pancreas. In the process of epithelial co-option, PDAC cells establish direct contact with adjacent non-neoplastic duodenal cells, and we speculate that the intercellular crosstalk between PDAC and enterocytes is key for tumor growth. Recent studies have begun to shed light on the molecular pathways that allow tumor cells to replace their non-malignant neighbors. These pathways involve tumor-host cell competition driven by Hippo or JNK signaling [27,28], and the secretion of Wnt antagonists by tumor cells to gain a competitive advantage [29]. It is remarkable that PDAC might be capable of adopting similar mechanisms outside of its host organ, and that the change in the mode of growth (destructive *vs*. replacement) is tightly connected to the cellular phenotype. Further studies are warranted to disentangle the underlying molecular pathways. However, a particular challenge is the fact that intestinal mimicry is infrequently observed in both humans and mice, limiting the number of specimens available to study the underlying mechanisms. It is unclear to what extent sampling bias might contribute to the scarcity of cases we found, given that assessing the duodenal mucosa for tumor cell integration is only feasible for a limited region of the mucosa. We hope that our smq-IHC data together with, for example, spatial transcriptomic analysis of FFPE tissue at single-cell resolution will help to identify key pathways of non-destructive PDAC growth in the future.

Together, our data define a real-life endpoint of the phenotypic plasticity of PDAC cells in humans. They strongly support a model in which basal-like *vs*. classical tumor subtypes are highly influenced by microenvironmental cues. The consistency of this phenomenon suggests that this and similar cohorts displaying duodenal invasion can be invaluable for deciphering the molecular underpinnings of PDAC subtype emergence orchestrated by the microenvironment.

## CRediT authorship contribution statement

**Benedek Bozóky:** Writing – original draft. **Carlos Fernández Moro:** Writing – original draft. **Carina Strell:** . **Natalie Geyer:** . **Rainer L. Heuchel:** . **J. Matthias Löhr:** . **Ingemar Ernberg:** Supervision. **Laszlo Szekely:** Supervision. **Marco Gerling:** Conceptualization, Writing – original draft. **Béla Bozóky:** Conceptualization.

## CRediT authorship contribution statement

**Benedek Bozóky:** Conceptualization, Writing – original draft, Formal analysis, Visualization. **Fernández Moro:** Conceptualization, Writing – original draft, Data curation, Formal analysis, Methodology, Supervision. **Carina Strell:** Formal analysis, Methodology, Writing – review & editing. **Natalie Geyer:** Visualization, Writing – review & editing. **Rainer L. Heuchel:** Resources, Formal analysis. **J. Matthias Löhr:** Supervision, Resources, Writing – review & editing, Funding acquisition. **Ingemar Ernberg:** Supervision, Writing – review & editing. **Laszlo Szekely:** Supervision, Formal analysis. **Marco Gerling:** Conceptualization, Writing – original draft, Resources, Supervision, Visualization, Project administration, Funding acquisition. **Béla Bozóky:** Formal analysis, Investigation, Methodology, Supervision, Data curation, Writing – review & editing.

## Declaration of Competing Interest

The authors declare that no conflicts of interest exist.

## Appendix Supplementary materials

Image, application 1

Image, application 2

Image, application 3
