# Supplementary material for: Stabilization of the classical phenotype upon integration of pancreatic cancer cells into the duodenal epithelium
Source: Neoplasia. 2021 Nov 16;23(12):1300–6. doi: 10.1016/j.neo.2021.11.007 (PMC8605302; doi:10.1016/j.neo.2021.11.007)
Supplement: Supplementary file 2 [file mmc2.pdf]

Supplementary Data

Stabilization of the classical phenotype upon integration of pancreatic cancer cells into the duodenal epithelium

Benedek Bozóky, Carlos Fernández Moro, Carina Strell, Natalie Geyer, Rainer L. Heuchel, J.-Matthias Löhr, Ingemar Ernberg, Laszlo Szekely, Marco Gerling & Béla Bozóky

Supplementary Figure 1

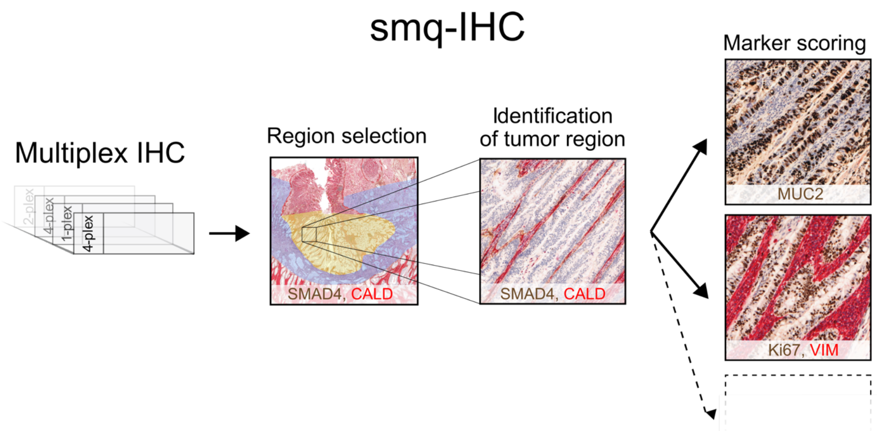

Supplementary Figure 1: Schematic of serial multiplex quantitative immunohistochemistry (smq-IHC). Consecutive sections are stained with different antibody combinations. IHC of SMAD4 and p53, as well as hematoxylin & eosin (H&E) stained sections guide the identification of tumor regions in the mucosa (yellow) and submucosa (blue in panel “Region selection”). Carefully matched regions of the consecutive sections are evaluated for protein expression, while SMAD4/p53 and H&E sections are continuously used to match the tumor regions in the mucosal and submucosal compartments. The percentage of positive cells in proportion of all tumor cells is evaluated. SMAD4: Mothers Against Decapentaplegic Homolog 4; p53: Tumor Protein 53; MUC5A: Mucin 5A; Ki67: Marker of Proliferation Ki-67.

Formatted: Font: Not Italic

Deleted: 1

Supplementary Table 1

Antigen	Epitope name	Main target cell	Clone	Dilution	Pretreat.*	Manufacturer-cat#
**CA125**	Cancer-antigen 125	PDAC cells [2]	OV185:1	1:100	H2	NCl-L-Ca125
**CA19-9**	Cancer-antigen 19-9	PDAC cells [3]	CA241:5:1:4	1:400	H2	NCL-L-CA19-9
**Caldesmon**	Caldesmon	smooth muscle cells [4]	H-CD	1:300	H2	Dako-M3557
**CD146**	Cluster of differentiation 146	*lamina propria* stroma and pericryptal mesenchymal cells [5]	UMAB154	1:100	H2	Origene-UM800051
**CD34**	Cluster of differentiation 34	fibrocytes/non-myofibroblastic stromal cells of the *lamina propria* [6,7]	QBEnd/10	1:50	H2	Dako-M7165
**CDX2**	caudal type homeobox 2	intestinal epithelial cells [8] and PDAC cells of classical type [9]	AMT28	1:25	H2	NCL-CDX2
**CEA(-m)**	Carcinoembryonic antigen - monoclonal	PDAC cells [2]	11-7	1:400	H2	Dako-M7072
**Chromogranin A**	Chromogranin A	enteroendocrine cells [10]	5H7	1:100	H1	NCL-CHROM-430
**CK5**	Cytokeratin 5	PDAC cells of the basal-like type [9]	XM26	1:100	H2	NCL-L-CK5
**CK7**	Cytokeratin 7	PDAC cells (pan-PDAC marker) [11]	RN7	1:200	H2	NCL-L-CK7-560
**CK17**	Cytokeratin 17	PDAC cells [2] of the basal-like type [9,12]	E3	1:25	H2	NCL-CK17
**CK20**	Cytokeratin 20	cells with intestinal differentiation [13] and classical type PDAC cells [9,12]	PW31	1:25	H2	NCL-L-CK20-561
**D2-40**	Podoplanin	desmoplastic stroma [14]	D2-40	1:50	H2	Dako-M3619
**HMGA2**	high-motility group A2	basal-like cells [9]	clone D17	1:400	n/a	Cell Signaling-#8179
**Ki67**	marker of proliferation Ki-67	cycling cells [15]	MIB-1	1:150	H2	Dako-M7240
**Maspin**	Maspin	PDAC cells [16]	EAW24	1:50	H2	NCL-Maspin
**MUC1**	Mucin 1	PDAC cells [17]	Ma695	1:50	H1	NCL-MUC1
**MUC2**	Mucin 2	intestinal goblet cells [18] and PDAC cells of classical type [9]	Ccp58	1:100	H2	NCL-MUC2
**MUC5AC**	Mucin 5AC	PDAC [2] of the classical type [9]	CLH2	1:50	H2	NCL-MUC-5Ac
**MUC6**	Mucin 6	PDAC cells [2]	CLH5	1:50	H2	NCL-MUC-6
**NGFR**	Nerve growth factor receptor (p75)	pancreatic stroma [19]	"Polyclonal"	1:200	H2	Atlas-HPA004765
**p53**	tumor protein p53	p53 protein [20]	Do-7	1:300	H1	NCL-L-P53-D07
**SMAD4**	Mothers against decapentaplegic homolog 4	SMAD4 protein [21]	B-8	1:300	H2	Santa Cruz-sc-7966
**Vimentin**	Vimentin	stromal cells and cells undergoing EMT [22,23]	V9	1:1500	H1	Dako-M0725
**WT1**	Wilms tumor 1 (WT1 transcription factor)	cells of the intestinal *lamina propria* [24]	6F-H2	1:100	H2	Dako-M3561

Supplementary Table 1: Antibodies used for serial multiplex quantitative immunohistochemistry.
***** "Pretreatment" refers to either "*H1*": Bond Epitope Retrieval Solution 1, citrate, 20 minutes, or "*H2*" = Bond Epitope Retrieval Solution 2, EDTA, 20 minutes; NCL: Novocastra/Leica. HMGA2 staining was performed manually, as previously reported[1].

Deleted:

Deleted: s

Deleted: were

Supplementary Table 2

Antigen	Number of cases	Mean mucosa (%)	Mean submucosa (%)	Min. mucosa (%)	Max. mucosa (%)	Min. submucosa (%)	Max. submucosa (%)
**CA125**	17	22.9	34.1	0	92.5	0	92.5
**Ca19-9**	20	63.6	70.2	0	92.5	0	92.5
**CDX2**	17	32.9	18.2	0	95	0	95
**CEA-m**	17	43.1	36.3	0	92.5	0	92.5
**CK17**	18	17.2	34.3	0	60	0	80
**CK20**	19	14.2	5.3	0	70	0	50
**CK7**	19	77.2	78.0	0	92.5	0	92.5
**Ki67**	15	42.0	29.7	20	60	10	60
**MUC1**	18	25.0	26.4	0	92.5	0	92.5
**MUC2**	19	7.6	0.8	0	60	0	15
**MUC5AC**	20	63.5	40.5	15	92.5	0	92.5
**MUC6**	16	14.1	10.3	0	60	0	92.5

Supplementary Table 2: Protein expression in the tumor cells as assessed by serial multiplex quantitative immunohistochemistry. Expression (mean, minimum [min.] and maximum [max.]) as percentage of positively stained tumor cells for each protein analyzed.

Supplementary References

1 Strell C, Norberg KJ, Mezheyeuski A, Schnittert J, Kuninty PR, Moro CF *et al.* Stroma-regulated HMGA2 is an independent prognostic marker in PDAC and AAC. *Br J Cancer* 2017; **117**: 65–77.

2 Fernández Moro C, Fernandez-Woodbridge A, Alistair D'souza M, Zhang Q, Bozoky B, Kandaswamy SV *et al.* Immunohistochemical Typing of Adenocarcinomas of the Pancreatobiliary System Improves Diagnosis and Prognostic Stratification. *PLoS One* 2016; **11**: e0166067.

3 Zapata M, Cohen C, Siddiqui MT. Immunohistochemical expression of SMAD4, CK19, and CA19-9 in fine needle aspiration samples of pancreatic adenocarcinoma: Utility and potential role. *Cytojournal* 2007; **4**: 13.

4 Eves R, Webb BA, Zhou S, Mak AS. Caldesmon is an integral component of podosomes in smooth muscle cells. *Journal of Cell Science* 2006; **119**: 1691–1702.

5 Signore M, Cerio AM, Boe A, Pagliuca A, Zaottini V, Schiavoni I *et al.* Identity and ranking of colonic mesenchymal stromal cells. *J Cell Physiol* 2012; **227**: 3291–3300.

6 Barth PJ, Westhoff CC. CD34+ fibrocytes: morphology, histogenesis and function. *Curr Stem Cell Res Ther* 2007; **2**: 221–227.

7 Stzepourginski I, Nigro G, Jacob J-M, Dulauroy S, Sansonetti PJ, Eberl G *et al.* CD34+ mesenchymal cells are a major component of the intestinal stem cells niche at homeostasis and after injury. *PNAS* 2017; **114**: E506–E513.

8 Saad RS, Ghorab Z, Khalifa MA, Xu M. CDX2 as a marker for intestinal differentiation: Its utility and limitations. *World J Gastrointest Surg* 2011; **3**: 159–166.

9 Chan-Seng-Yue M, Kim JC, Wilson GW, Ng K, Figueroa EF, O'Kane GM *et al.* Transcription phenotypes of pancreatic cancer are driven by genomic events during tumor evolution. *Nat Genet* 2020; **52**: 231–240.

10 Campana D, Nori F, Piscitelli L, Morselli-Labate AM, Pezzilli R, Corinaldesi R *et al.* Chromogranin A: Is It a Useful Marker of Neuroendocrine Tumors? *JCO* 2007; **25**: 1967–1973.

11 Duval JV, Savas L, Banner BF. Expression of cytokeratins 7 and 20 in carcinomas of the extrahepatic biliary tract, pancreas, and gallbladder. *Arch Pathol Lab Med* 2000; **124**: 1196–1200.

12 Moffitt RA, Marayati R, Flate EL, Volmar KE, Loeza SGH, Hoadley KA *et al.* Virtual microdissection identifies distinct tumor- and stroma-specific subtypes of pancreatic ductal adenocarcinoma. *Nat Genet* 2015; **47**: 1168–1178.

13 Perysinakis I, Minaidou E, Leontara V, Mantas D, Sotiropoulos GC, Tsipras H *et al.* Differential Expression of β-Catenin, EGFR, CK7, CK20, MUC1, MUC2, and CDX2 in Intestinal and Pancreatobiliary-Type Ampullary Carcinomas. *Int J Surg Pathol* 2017; **25**: 31–40.

14 Shindo K, Aishima S, Ohuchida K, Fujiwara K, Fujino M, Mizuuchi Y *et al.* Podoplanin expression in cancer-associated fibroblasts enhances tumor progression of invasive ductal carcinoma of the pancreas. *Mol Cancer* 2013; **12**: 168.

15 Scholzen T, Gerdes J. The Ki-67 protein: From the known and the unknown. *Journal of Cellular Physiology* 2000; **182**: 311–322.

16 Nash JW, Bhardwaj A, Wen P, Frankel WL. Maspin is useful in the distinction of pancreatic adenocarcinoma from chronic pancreatitis: a tissue microarray based study. *Appl Immunohistochem Mol Morphol* 2007; **15**: 59–63.

17 Hinoda Y, Ikematsu Y, Horinochi M, Sato S, Yamamoto K, Nakano T *et al.* Increased expression of MUC1 in advanced pancreatic cancer. *J Gastroenterol* 2003; **38**: 1162–1166.

18 Pelaseyed T, Bergström JH, Gustafsson JK, Ermund A, Birchenough GMH, Schütte A *et al.* The mucus and mucins of the goblet cells and enterocytes provide the first defense line of the gastrointestinal tract and interact with the immune system. *Immunol Rev* 2014; **260**: 8–20.

19 Xue R, Jia K, Wang J, Yang L, Wang Y, Gao L *et al.* A Rising Star in Pancreatic Diseases: Pancreatic Stellate Cells. *Front Physiol* 2018; **9**. doi:10.3389/fphys.2018.00754.

20 Lundin J, Nordling S, von Boguslawsky K, Roberts PJ, Haglund C. Prognostic value of immunohistochemical expression of p53 in patients with pancreatic cancer. *Oncology* 1996; **53**: 104–111.

21 Tascilar M, Skinner HG, Rosty C, Sohn T, Wilentz RE, Offerhaus GJA *et al.* The SMAD4 Protein and Prognosis of Pancreatic Ductal Adenocarcinoma. *Clin Cancer Res* 2001; **7**: 4115–4121.

22 Maehira H, Miyake T, Iida H, Tokuda A, Mori H, Yasukawa D *et al.* Vimentin Expression in Tumor Microenvironment Predicts Survival in Pancreatic Ductal Adenocarcinoma: Heterogeneity in Fibroblast Population. *Ann Surg Oncol* 2019; **26**: 4791–4804.

23 Vuoriluoto K, Haugen H, Kiviluoto S, Mpindi J-P, Nevo J, Gjerdrum C *et al.* Vimentin regulates EMT induction by Slug and oncogenic H-Ras and migration by governing Axl expression in breast cancer. *Oncogene* 2010; **30**: 1436–1448.

24 Parenti R, Salvatorelli L, Musumeci G, Parenti C, Giorlandino A, Motta F *et al.* Wilms' tumor 1 (WT1) protein expression in human developing tissues. *Acta Histochem* 2015; **117**: 386–396.
