# Supplementary material for: Stabilization of the classical phenotype upon integration of pancreatic cancer cells into the duodenal epithelium
Source: Neoplasia. 2021 Nov 16;23(12):1300–6. doi: 10.1016/j.neo.2021.11.007 (PMC8605302; doi:10.1016/j.neo.2021.11.007)
Supplement: Supplementary file 3 [file mmc3.pdf]

Stabilization of the classical phenotype upon integration of pancreatic cancer cells into the duodenal epithelium

Benedek Bozóky[1,2,], Carlos Fernández Moro[3,4], Carina Strell[5,6], Natalie Geyer[7], Rainer L. Heuchel[8], J.-Matthias Löhr[1,8], Ingemar Ernberg[2], Laszlo Szekely[3,4], Marco Gerling[1,7,#,*] & Béla Bozóky[3,*]

Addresses:

1. Theme Cancer, Karolinska University Hospital, 17176 Solna, Sweden
2. Department of Microbiology, Tumor, and Cell Biology, Karolinska Institutet, Solnavägen 9, 17165 Solna, Sweden
3. Department of Clinical Pathology/Cytology, Karolinska University Hospital, 14186 Huddinge, Sweden
4. Department of Laboratory Medicine, Division of Pathology, Karolinska Institutet, 14186 Huddinge, Sweden
5. Department of Oncology-Pathology, Karolinska Institutet, 17164 Solna, Sweden
6. Department of Immunology, Genetics and Pathology, Uppsala University, 75185 Uppsala, Sweden
7. Department of Biosciences and Nutrition, Karolinska Institutet, 14183 Huddinge, Sweden
8. Department of Clinical Science, Intervention and Technology (CLINTEC), Karolinska Institutet, 14186 Huddinge, Sweden.

[*]shared authorship

[#]To whom correspondence should be addressed:

Dr. med. Marco Gerling
Karolinska Institutet
Department of Biosciences and Nutrition
Hälsovägen 7
141 83 Huddinge
Sweden
marco.gerling@ki.se

Conflicts of Interest: The authors declare that no conflicts of interest exist.

Highlights

- Pancreatic cancer cells that infiltrate the duodenum mimic intestinal epithelial cells, revert to non-destructive growth, and co-opt the native basement membrane without induction of stromal desmoplasia.
- Intramucosal PDAC location is strongly coupled to the classical phenotype and intestinal traits (higher MUC5AC, MUC2, CK20 and lower CK17, CA125 expression).
- Intratumoral heterogeneity is linked to specific tissue compartments, which shape phenotype plasticity of PDAC cells in humans.

Deleted: -

Abstract (word count: 250)

Introduction: Pancreatic ductal adenocarcinoma (PDAC) is one of the most aggressive solid tumors. Based on transcriptomic classifiers, basal-like and classical PDAC subtypes have been defined that differ in prognosis. Cells of both subtypes can coexist in individual tumors; however, the contribution of either clonal heterogeneity or microenvironmental cues to subtype heterogeneity is unclear. Here, we report the spatial tumor phenotype dynamics in a cohort of patients in whom PDAC infiltrated the duodenal wall, and identify the duodenal epithelium as a distinct PDAC microniche.

Materials and Methods: We used serial multiplex quantitative immunohistochemistry (smq-IHC) for 24 proteins to phenotypically chart PDAC tumor cells in patients whose tumors infiltrated the duodenal epithelium. Additionally, we used a genetically engineered mouse model to study the PDAC cell phenotype in the small intestinal epithelium in a controlled genetic background.

Result: We show that pancreatic cancer cells revert to non-destructive growth upon integration into the duodenal epithelium, where they adopt traits of intestinal cell differentiation, associated with phenotypical stabilization of the classical subtype. The integrated tumor cells replace epithelial cells in an adenoma-like manner, as opposed to invasive growth in the submucosa. Finally, we show that this phenomenon is shared between species, by confirming duodenal integration and phenotypic switching in a genetic PDAC mouse model.

Discussion: Our results identify the duodenal epithelium as a distinct PDAC microniche and tightly link microenvironmental cues to cancer transcriptional subtypes. The phenomenon of “intestinal mimicry” provides a unique opportunity for the systematic investigation of microenvironmental influences on pancreatic cancer plasticity.

Key words: Pancreatic cancer, transcriptome subtypes, tumor microenvironment, local invasion, intestinal mimicry

Deleted: .
Deleted: However
Deleted: 3
Deleted: different
Deleted: We
Deleted: phenotype of
Deleted: s
Deleted:
Deleted: Integrated
Deleted: into the duodenal epithelium,
Deleted: pancreas
Deleted: I

Introduction

PDAC is one of the most lethal tumors with a five-year survival rate of less than 9%[1]. Although clinically perceived as uniformly aggressive, PDAC subtypes with varying clinical outcomes can be defined based on transcriptional profiling[2–6]. Classifications distinguish two major subtypes, "classical" and "basal-like", although intermediate states and less common subtypes exist[2]. Basal-like tumors are associated with a worse prognosis[2–4,6]. While subtyping based on bulk transcriptomic data is prognostically valuable, cells with both a basal-like and a classical phenotype co-exist in individual tumors, as revealed by single-cell RNA sequencing[2]. Genetic changes, such as allelic imbalances of mutant *KRAS*, contribute to this intratumor heterogeneity[2,7]; however, mouse models and organoid co-cultures have demonstrated a central role for the microenvironment in shaping the PDAC tumor cell phenotype[8,9], and there is strong support from experimental studies that the non-malignant microenvironment can reprogram malignant cells to a normal-like behavior[10,11]. In human PDAC, cancer cell states have recently been linked to adjacent fibroblast subtypes[12]. Nevertheless, the full extent to which microenvironmental cues shape tumor cell phenotypes remains unclear.

Routine pathological assessment regularly reveals morphological heterogeneity in pancreatic tumors[13]. In clinical cases where a small intestinal mass is biopsied endoscopically, PDAC can occasionally be misdiagnosed as an intestinal neoplasm, or even be mistaken for reactive small intestinal changes. This rare, but clinically important phenomenon – that poses diagnostic difficulties – has previously been termed "intestinal mimicry", and an immunohistochemical marker panel has been proposed to improve PDAC diagnosis based on intestinal biopsies[14,15].

The ability of PDAC cells to escape the pathologist's eye once settled in the duodenal epithelium suggests strong phenotypical changes in the tumor cells, while leaving the underlying stroma relatively intact. This is remarkable, given the destructive mode of growth and the strong desmoplastic stromal reaction that otherwise characterize PDAC[13].

Based on the emerging PDAC subtypes, we have studied in detail the changes relating to intestinal mimicry that occur in the tumor cell phenotype upon switching location from the pancreas to the small intestine. We have systematically mapped tumor cell phenotype dynamics in a unique cohort of PDAC patients with duodenal

Deleted: a
Deleted: of
Deleted: here
Deleted: in intestinal mimicry, i.e.
Deleted: of the tumor cells
Deleted: the

infiltration, collected over more than a decade of routine pathological diagnostics at a large tertiary care center specializing in pancreatic resections. We found that PDAC cells in the small intestinal epithelium revert to non-destructive growth, and switch to a purely classical phenotype upon epithelial integration. In the duodenal epithelium, protein expression of PDAC cells mimics that of small intestinal enterocytes, while the stroma retains its small intestinal identity devoid of desmoplasia. For the first time, our results link the small intestinal microenvironment to defined shifts in PDAC tumor cell subtypes. Together, they suggest that intestinal mimicry provides the remarkable – yet largely overlooked – possibility of studying cancer cell differentiation towards a less aggressive, near-normal phenotype in relation to microenvironmental cues and within spatially defined tissue compartments.

Material and Methods

Patients

Patients with duodenal infiltration of primary PDAC were identified by a retrospective search in the pathology archive database of the Karolinska University Hospital, Huddinge, Sweden, and through routine diagnostic pathology between 2008 and 2020. In all cases, the tumor epicenter was located in the pancreas and all tumors were classified as PDAC histologically and by the local multidisciplinary tumor board.

Cases in which infiltration of PDAC cells into the small intestine was described in the pathology report, or for which the participating pathologists (CFM, Béla B, LS) noted this phenomenon, were selected and systematically reassessed based on available hematoxylin and eosin (H&E) staining and immunohistochemistry (IHC). Clinical data were obtained by retrospective chart review.

Serial multiplex quantitative immunohistochemistry

Serial multiplex quantitative immunohistochemistry (smq-IHC) was performed as described previously[16]. Briefly, formalin-fixed paraffin-embedded (FFPE) samples were cut to a thickness of 4 μm and stained on an automated stainer (BOND-MAX, Leica Biosystems, Germany) as part of the diagnostic routine in a clinically accredited histology lab. Staining procedures have been described previously[16]. Antibodies and staining protocols are presented in **Supplementary Table 1**.

Deleted: centre
Deleted: specialized
Deleted: on
Deleted: find
Deleted: display a
Deleted: Our results, f
Deleted: a
Deleted: Hematoxylin
Deleted: Eosin
Deleted: s

For quantification, H&E stains as well as IHC for Mothers against decapentaplegic homolog 4 (SMAD4) and Tumor protein 53 (p53) guided the identification of tumor regions and individual cells, which were then manually matched to corresponding regions in serial sections, while SMAD4/p53 stains were used to navigate through the sections (**Supplementary Figure 1**). All identifiable tumor cells in the mucosa and submucosa of one representative paraffin block were included in the quantification process. The average areas on which the calculations are based were: 15.3 mm^2 (range 3.7–36.6 mm^2) for the mucosa and 37.2 mm^2 (range 7.0–153.9 mm^2) for the submucosa, respectively.

Quantification was achieved by calculating the percentage of positive cells with respect to all tumor cells in the two separate regions, i.e. mucosa and submucosa.

For all antibody stains, at least n = 10 cases were included for the final assessment, based on staining quality and availability. For all quantified antibody stains, statistics are based on the evaluation of at least n= 15 matched mucosa/submucosa pairs.

This study was approved by the responsible Ethical Review Board (no. 2020-06115, *Etikprövningsmyndigheten* and 2015/259-31/2, *Etikprövningsnämnd*, Sweden).

Mice

FFPE tissue sections from a cohort of *Kras*$^{LSL-G12D/+}$;*Trp53*$^{LSL-R172H;+}$;*Pdx1-Cre* (KPC) mice[17] described previously[18] were analyzed for the presence of duodenal invasion based on available H&E sections. One animal was identified where PDAC cells had infiltrated the duodenum; FFPE sections from this mouse were assessed for expression of the high-motility group AT-hook 2 (HMGA2) protein by immunohistochemistry, as described previously[18]. The antibody used is listed in **Supplementary Table 1**. Animal experiments were approved by the Swedish Board of Agriculture, Sweden, Nr. S31/15 (*Stockholms Södra Djurförsöksetiska Nämnd*).

Results

Consistent morphological changes in PDAC cells in the duodenum

A total of n = 20 patients in whom PDAC cells had infiltrated the entire thickness of the duodenal wall were identified. All patients (n = 8 females, n = 12 males, aged 64–82

Deleted: ings
Deleted: of interest
Deleted: corresponding
Deleted: ing
Deleted: resultss
Deleted: also continuously
Deleted: done
Deleted: per
Deleted: defined
Deleted: stainings
Deleted: stainings
Deleted: (**Figure 1K and L**)
Deleted:
Deleted: Antibodies and staining protocols are presented in **Supplementary Table 1**.¶
Deleted: e
Deleted: ,
Deleted: the
Deleted: protein
Deleted: A 2
Deleted: *of*

years, median age 71.8 years) underwent resection according to Whipple at Karolinska University Hospital, Stockholm, Sweden. A total of n = 19 patients passed away during the observation time, median overall survival was n = 559 days after operation (range n = 3 to n = 2460 days). One patient was alive when data collection was completed in January 2021.

In patients where PDAC cells had infiltrated the duodenal epithelium, we observed consistent morphological changes that accompanied tumor cell integration into the epithelial layer (**Figure 1A**). In all cases, the mucosal architecture was strikingly preserved, and the epithelial lining exhibited either normal morphology or mild reactive atypia, interspersed with areas of columnar epithelium with a dysplastic/neoplastic appearance, in line with the previously recognized diagnostic challenge[15]. To confirm unequivocally that the neoplastic cells in the duodenal mucosa were of pancreatic origin, rather than reactive intestinal cells secondary to PDAC infiltration into the submucosa, we used two independent immunohistochemical markers, SMAD4 and p53; SMAD4 is lost in approximately half of all pancreatic cancer cases[19]; while *TP53* mutations, which lead to accumulation of mutant p53 protein, are detectable in 50% of patients, independently of alterations in *SMAD4*[19]. Both markers confirmed the seamless integration of PDAC cells into the SMAD4^{+ve}/p53$^{-ve/low}$ duodenal epithelium, without destruction of the mucosal architecture (**Figure 1B**).

Intramucosal PDAC cells were well differentiated and polarized, in contrast to the submucosa, which harbored pleomorphic cancer cells and irregular glands, consistent with primary PDAC histomorphology. A hallmark of PDAC is its desmoplastic stroma, in which tumor glands are embedded[12]. Notably, IHC for the desmoplasia marker, podoplanin (D2-40)[20], revealed no desmoplasia of the subepithelial stroma adjacent to mucosal cancer cell integration, while desmoplasia was present in the submucosa (**Figure 1C**). In contrast, protein expression of CD146 and WT1 (**Figure 1D**), markers of the intestinal *lamina propria*[21,22], was preserved in regions adjacent to intraepithelial tumor cells.

Stabilization of the classical phenotype upon epithelial integration

The diverging phenotypes of PDAC cells in mucosal *vs.* submucosal locations implied a high degree of location-dependency, suggesting that the local microenvironment is a major contributor to the tumor cell phenotype. To quantify these phenotypic

differences, we used smq-IHC to assess a panel of 12 protein markers, selected to identify tumor cell characteristics, and to approximate the transcriptional subtypes[2] (see also **Supplementary Table 2**), as previous studies had shown that condensed marker panels can differentiate between basal-like and classical subtypes with high accuracy[23]. The panel comprised intestinal as well as pancreatobiliary differentiation markers (CK20, CDX2, MUC2, MUC1, MUC5AC), all of which are included in the classical transcriptional profile[2], a pancreatobiliary marker specific to the basal-like profile (CK17), general markers for PDAC cells (CK7, MUC6) and tumor-specific glycoproteins (CA19-9, CA125, and CEA), together with Ki67 to assess proliferation.

The smq-IHC results revealed significantly reduced expression of the basal-like marker, CK17, along with CA125, in the mucosa *vs.* submucosa. In contrast, the expression of the classical/intestinal markers, MUC5AC, CK20, and MUC2 was significantly increased in the mucosa compared to the submucosa (**Figures 2A & B**, **Supplementary Table 2**), supporting a strong phenotypic switch away from basal-like towards classical differentiation following intramucosal integration, which was accompanied by enhanced Ki67 positivity (**Figure 2A**).

Murine PDAC recapitulates phenotypic plasticity

The KPC mouse model[17] closely recapitulates PDAC morphology, driven by mutations in *Kras* and *Tp53* under the control of a pancreas-specific promoter (*Pdx1*). To assess whether small intestinal infiltration of PDAC leads to morphological changes similar to those in humans, we analyzed tissues from n = 6 KPC mice with PDAC. We identified one animal, in which PDAC cells infiltrated the small intestine to the level of the epithelium. Morphological changes were similar to those observed in humans, such that the tumor cells in the epithelial layer were polarized and morphologically mimicked enterocytes (**Figure 3A**). Next, we assessed the expression of HMGA2, a transcriptional marker for basal-like tumor cells in human PDAC[2], that is also expressed in murine KPC tumors and for which staining of murine tissue has been established previously[18]. In concordance with human PDAC, HMGA2 was downregulated in murine PDAC cells located in the epithelial layer (**Figure 3B**) compared to PDAC cells in the submucosa, consistent with the attenuation of the basal-like phenotype upon intestinal integration.

Deleted: serial multiplex quantitative immunohistochemistry (…
Deleted:)
Deleted: (see also **Supplementary Table 2**
Deleted: in
Deleted: similar
Deleted: as
Deleted: location
Deleted: as
Deleted: (**Figure 3B**)

Discussion

The tumor microenvironment influences cancer development and progression, exerting both cancer-promoting and restraining effects. The extent to which tumor location – and hence the spatial relationship of tumor cells to their specific microenvironment – shapes the PDAC cell phenotype is unclear. Here, we show that the integration of PDAC cells into the duodenal mucosa is associated with a quantifiable phenotypic shift towards intestinal differentiation, identifying the duodenal epithelium as a specific PDAC microniche (**Figure 4**). Location-dependent morphological changes are accompanied by a loss of basal-like subtype markers in favor of classical subtype markers, corresponding to a switch towards a less aggressive molecular phenotype, and strong intestinal cell-like differentiation of the integrated tumor cells[2–4,6]. Interestingly, mucosal PDAC cells were cycling (Ki67^{+ve}) at higher levels than submucosal tumor cells, suggesting the uncoupling of differentiation from proliferation. While the increase in Ki67 positivity in conjunction with a less aggressive phenotype may appear counterintuitive, results from studies on the prognostic value of Ki67 expression in PDAC have been mixed[24,25]. The intestinal mucosa is a highly proliferative tissue that renews every five days[26] and hence, the increase in proliferative activity may be interpreted as part of the alignment of PDAC cells with their epithelial location.

Our results establish that PDAC cells integrate into the epithelial compartment of the duodenum, where they progress in an *in situ*-like manner. Importantly, for the first time, we provide evidence that this phenomenon is connected to the distinct tumor subtypes. Non-destructive growth in the epithelium requires PDAC cells to respect the native basement membrane, and it does not induce stromal desmoplasia; hence, it differs significantly from tumor growth in the pancreas. In the process of epithelial co-option, PDAC cells establish direct contact with adjacent non-neoplastic duodenal cells, and we speculate that the intercellular crosstalk between PDAC and enterocytes is key for tumor growth. Recent studies have begun to shed light on the molecular pathways that allow tumor cells to replace their non-malignant neighbors. These pathways involve tumor-host cell competition driven by Hippo or JNK signaling[27,28], and the secretion of Wnt antagonists by tumor cells to gain a competitive advantage[29]. It is remarkable that PDAC might be capable of adopting similar mechanisms outside of its host organ, and that the change in the mode of growth (destructive *vs.* replacement) is tightly connected to the cellular. Further studies are warranted to

Deleted:
Deleted: (Ki67^{+ve})
Deleted: (Ki67^{+ve})
Deleted: of
Deleted:
Deleted:
Deleted: adaptation
Deleted: to
Deleted: .
Deleted: to
Deleted: , which
Deleted: and
Deleted:
Deleted: could be able to
Deleted: is
Deleted: uch
Deleted: their
Deleted: transcriptomic
Deleted: phenotype

disentangle the underlying molecular pathways. However, a particular challenge is the fact that intestinal mimicry is infrequently observed in both humans and mice, limiting the number of specimens available to study the underlying mechanisms. It is unclear to what extent sampling bias might contribute to the scarcity of cases we found, given that assessing the duodenal mucosa for tumor cell integration is only feasible for a limited region of the mucosa. We hope that our smq-IHC data together with, for example, spatial transcriptomic analysis of FFPE tissue at single-cell resolution will help to identify key pathways of non-destructive PDAC growth in the future.

Together, our data define a real-life endpoint of the phenotypic plasticity of PDAC cells in humans. They strongly support a model in which basal-like *vs*. classical tumor subtypes are highly influenced by microenvironmental cues. The consistency of this phenomenon suggests that this and similar cohorts displaying duodenal invasion can be invaluable for deciphering the molecular underpinnings of PDAC subtype emergence orchestrated by the microenvironment.

Deleted: ;
Deleted: h
Deleted: towards this end
Deleted: since
Deleted: possible
Deleted: s
Deleted: ing
Deleted: tightly

Acknowledgements

Marco Gerling's research group is supported by The Swedish Research Council (project nr. 2018-02023), The Swedish Society for Medical Research, the Åke Wiberg Foundation, the Jeansson Foundation and the Karolinska Institute. Ingemar Ernberg's laboratory is supported by The Swedish Cancer Society. The Pancreatic Cancer Research Laboratory (JML, RH) is supported by The Swedish Research Council and The Swedish Cancer Society. We are thankful for the support provided by the histological and immunohistochemical laboratories, Karolinska University Hospital. We thank Rune Toftgård and Arne Östman for comments on the manuscript.

Author contributions: Benedek B, Béla B, MG, CF, and CS generated and analyzed data from human samples; LS and IE advised on study design; RLH and JML contributed and analyzed mouse samples, Béla B and MG conceived the study, Benedek B, CFM and MG wrote the manuscript, All authors commented on the manuscript.

Deleted: ,

Deleted: ,

Figure Legends

Figure 1. Histology and tumor cell identification in duodenal invasion of pancreatic ductal adenocarcinoma (PDAC). A) Hematoxylin & eosin (H&E) staining of PDAC cells that have invaded and integrated into the duodenal mucosa. “N” indicates non-neoplastic duodenal epithelium. Scale bar: 1 mm. **B)** Left panel: Representative immunohistochemistry (IHC) for SMAD4 in a PDAC case with genetic loss of *SMAD4* shows intestinal villi lined by SMAD4-negative PDAC cells (asterisks) compared to adjacent small intestinal epithelial cells positive for SMAD4 expression (arrows). Right panel: Representative image of p53 IHC in a PDAC case with accumulation of p53 protein due to *TP53* mutation; note regions of p53-positive PDAC cells (asterisks) adjacent to p53-negative intestinal epithelial cells (arrows). Scale bar: 200 µm for both panels. **C)** Immunohistochemistry (IHC) for the indicated proteins illustrating overexpression of the desmoplasia marker D2-40 (podoplanin) in the submucosa compared to the mucosa; note that in this quadruple staining, the brown stain identifies both D2-40 (stromal) and p53 (epithelial) expression, and red identifies both caldesmon (Cald, stromal) and SMAD4 (epithelial) expression; asterisks indicate stroma. Note that the rare D2-40 positive structures visible in the mucosa represent lymphatic endothelial cells (dark brown, examples indicated with arrows). **D)** IHC for the lamina propria marker, CD146 (left panel), shows preserved expression in areas of intraepithelial tumor integration (arrows). IHC for another lamina propria marker, WT1 (right panel), also shows preserved expression in areas of tumor cell epithelial integration (arrows). Representative stainings of $n \geq 10$ cases. Scale bar: 200 µm (applies to C and D). All IHC counterstained with hematoxylin. Multiplex staining combinations are indicated in panels, text color denotes color of chromogen used to visualize protein expression.

Figure 2. Phenotypic shift of pancreatic cancer cells upon integration into the duodenal mucosa. A) Volcano plot showing significant findings in red (higher in mucosa) and blue (higher in submucosa) out of a total of $n = 12$ markers included in the analysis. Data based on results from Wilcoxon matched-pairs signed rank test, multiple test correction with two-stage step-up method (Benjamini, Hochberg, Yekutieli) FDR < 0.05. **B)** Differential protein expression in mucosal vs. submucosal tumor cells for MUC5AC, MUC2/MUC1, CK20/CK5, and WT1/CA125. Rightmost three

Deleted: E
Deleted: N:
Moved down [1]: Scale bar: 200 µm.
Deleted: PDAC cells,
Deleted: cells (
Deleted: ,
Moved (insertion) [1]
Deleted: , desmoplasia marker
Deleted: these
Deleted: s
Deleted: marks
Deleted: marks
Deleted: in the submucosalamina propria,
Deleted: H
Deleted: fdr
Deleted: Ki67

images from the same patient case, for which MUC5AC was not available (leftmost patient). Scale bar: 200 µm, applies to all images in (B); representative staining of n ≥ 15 cases. Note that multiplex immunohistochemistry was performed for some markers, but not all markers were included in quantitative analysis (e.g. WT1). Asterisks indicate mucosa, arrows indicate areas of tumor cells that have integrated into the epithelium.

Figure 3. Intestinal mimicry in a genetic mouse model of pancreatic cancer. A) Hematoxylin & eosin staining of murine small intestine infiltrated by tumor cells driven by mutations in *KRAS* and *P53* ($Kras^{LSL-G12D/+}$;$Trp53^{LSL-R172H;+}$;*Pdx1-Cre* mice, KPC). "N" indicates a region of normal small intestinal epithelium, "PDAC mucosa" indicates areas where tumor cells have integrated into the epithelial layer of the intestine; tumor cells are identified based on morphology, indicated by arrows in magnified lower panel. **B)** Immunohistochemistry for high mobility group AT-hook 2 (HMGA2) protein. HMGA2 is lost in areas of intestinal epithelial infiltration, while it is expressed in the submucosa; asterisk and arrow indicate extramucosal invasion and mucosal integration of PDAC cells, respectively. Scale bars: 250 µm for upper/lower panels of A and B, 100 µm for lower panels of A and B.

Figure 4. Illustration of the phenotypic switch of pancreatic cancer cells in the duodenum. Both basal-like and classical phenotypes co-exist in the submucosa. Cancer cells that integrate into the duodenal epithelium switch to a classical phenotype, recapitulate intestinal epithelial cell characteristics, respect the basement membrane, and grow in an adenoma-like manner.

Deleted: (e.g. Ki67 together with vimentin in the left panel),
Deleted: o
Deleted: Arrow
Deleted: s"N"
Deleted: s
Deleted: magnification
Deleted: (
Deleted:)
Deleted: abundantly
Deleted: abundantly in the tumor center in the pancreas (n = 6 mice with advanced tumors analyzed),
Deleted: submucosal
Deleted: /
Deleted: 100 µm for both (A) and (B) (
Deleted: scale bar applies to both lower panel images).
Deleted: **al mucosa**

References

1 Siegel RL, Miller KD, Jemal A. Cancer statistics, 2020. *CA: A Cancer Journal for Clinicians* 2020; **70**: 7–30.

2 Chan-Seng-Yue M, Kim JC, Wilson GW, Ng K, Figueroa EF, O'Kane GM *et al.* Transcription phenotypes of pancreatic cancer are driven by genomic events during tumor evolution. *Nat Genet* 2020; **52**: 231–240.

3 Moffitt RA, Marayati R, Flate EL, Volmar KE, Loeza SGH, Hoadley KA *et al.* Virtual microdissection identifies distinct tumor- and stroma-specific subtypes of pancreatic ductal adenocarcinoma. *Nat Genet* 2015; **47**: 1168–1178.

4 Maurer C, Holmstrom SR, He J, Laise P, Su T, Ahmed A *et al.* Experimental microdissection enables functional harmonisation of pancreatic cancer subtypes. *Gut* 2019; **68**: 1034–1043.

5 Bailey P, Chang DK, Nones K, Johns AL, Patch A-M, Gingras M-C *et al.* Genomic analyses identify molecular subtypes of pancreatic cancer. *Nature* 2016; **531**: 47–52.

6 Puleo F, Nicolle R, Blum Y, Cros J, Marisa L, Demetter P *et al.* Stratification of Pancreatic Ductal Adenocarcinomas Based on Tumor and Microenvironment Features. *Gastroenterology* 2018; **155**: 1999-2013.e3.

7 Mueller S, Engleitner T, Maresch R, Zukowska M, Lange S, Kaltenbacher T *et al.* Evolutionary routes and KRAS dosage define pancreatic cancer phenotypes. *Nature* 2018; **554**: 62–68.

8 Sahai E, Astsaturov I, Cukierman E, DeNardo DG, Egeblad M, Evans RM *et al.* A framework for advancing our understanding of cancer-associated fibroblasts. *Nature Reviews Cancer* 2020; **20**: 174–186.

9 Liu X, Gündel B, Li X, Liu J, Wright A, Löhr M *et al.* 3D heterospecies spheroids of pancreatic stroma and cancer cells demonstrate key phenotypes of pancreatic ductal adenocarcinoma. *Transl Oncol* 2021; **14**: 101107.

10 Bissell MJ, Radisky D. Putting tumours in context. *Nature Reviews Cancer* 2001; **1**: 46–54.

11 Ricca BL, Venugopalan G, Furuta S, Tanner K, Orellana WA, Reber CD *et al.* Transient external force induces phenotypic reversion of malignant epithelial structures via nitric oxide signaling. *eLife* 2018; **7**: e26161.

12 Ligorio M, Sil S, Malagon-Lopez J, Nieman LT, Misale S, Di Pilato M *et al.* Stromal Microenvironment Shapes the Intratumoral Architecture of Pancreatic Cancer. *Cell* 2019; **178**: 160-175.e27.

13 Verbeke C. Morphological heterogeneity in ductal adenocarcinoma of the pancreas – Does it matter? *Pancreatology* 2016; **16**: 295–301.

14 Campbell F, Verbeke CS. *Pathology of the pancreas: a practical approach*. Springer Science & Business Media, 2013.

15 Sopha SC, Gopal P, Merchant NB, Revetta FL, Gold DV, Washington K *et al.* Diagnostic and therapeutic implications of a novel immunohistochemical panel detecting duodenal mucosal invasion by pancreatic ductal adenocarcinoma. *Int J Clin Exp Pathol* 2013; **6**: 2476–2486.

16 Fernández Moro C, Fernandez-Woodbridge A, Alistair D'souza M, Zhang Q, Bozoky B, Kandaswamy SV *et al.* Immunohistochemical Typing of Adenocarcinomas of the Pancreatobiliary System Improves Diagnosis and Prognostic Stratification. *PLoS One* 2016; **11**: e0166067.

17 Hingorani SR, Wang L, Multani AS, Combs C, Deramaudt TB, Hruban RH *et al.* Trp53R172H and KrasG12D cooperate to promote chromosomal instability and widely metastatic pancreatic ductal adenocarcinoma in mice. *Cancer Cell* 2005; **7**: 469–483.

18 Strell C, Norberg KJ, Mezheyeuski A, Schnittert J, Kuninty PR, Moro CF *et al.* Stroma-regulated HMGA2 is an independent prognostic marker in PDAC and AAC. *Br J Cancer* 2017; **117**: 65–77.

19 Smith RA, Tang J, Tudur-Smith C, Neoptolemos JP, Ghaneh P. Meta-analysis of immunohistochemical prognostic markers in resected pancreatic cancer. *British Journal of Cancer* 2011; **104**: 1440–1451.

20 Shindo K, Aishima S, Ohuchida K, Fujiwara K, Fujino M, Mizuuchi Y *et al.* Podoplanin expression in cancer-associated fibroblasts enhances tumor progression of invasive ductal carcinoma of the pancreas. *Mol Cancer* 2013; **12**: 168.

21 Signore M, Cerio AM, Boe A, Pagliuca A, Zaottini V, Schiavoni I *et al.* Identity and ranking of colonic mesenchymal stromal cells. *J Cell Physiol* 2012; **227**: 3291–3300.

22 Parenti R, Salvatorelli L, Musumeci G, Parenti C, Giorlandino A, Motta F *et al.* Wilms' tumor 1 (WT1) protein expression in human developing tissues. *Acta Histochem* 2015; **117**: 386–396.

23 O'Kane GM, Grünwald BT, Jang G-H, Masoomian M, Picardo S, Grant RC *et al.* GATA6 Expression Distinguishes Classical and Basal-like Subtypes in Advanced Pancreatic Cancer. *Clin Cancer Res* 2020; **26**: 4901–4910.

24 Striefler JK, Sinn M, Pelzer U, Jühling A, Wislocka L, Bahra M *et al.* P53 overexpression and Ki67-index are associated with outcome in ductal pancreatic adenocarcinoma with adjuvant gemcitabine treatment. *Pathology - Research and Practice* 2016; **212**: 726–734.

25 Stanton KJ, Sidner RA, Miller GA, Cummings OW, Schmidt CM, Howard TJ *et al.* Analysis of Ki-67 antigen expression, DNA proliferative fraction, and survival in resected cancer of the pancreas. *The American Journal of Surgery* 2003; **186**: 486–492.

26 Barker N, Van De Wetering M, Clevers H. The Intestinal Stem Cell. *Genes Dev* 2008; **22**: 1856–1864.

27 Suijkerbuijk SJE, Kolahgar G, Kucinski I, Piddini E. Cell Competition Drives the Growth of Intestinal Adenomas in Drosophila. *Curr Biol* 2016; **26**: 428–438.

28 Garcia AK, Fumagalli A, Le HQ, Sansom OJ, Rheenen J van, Suijkerbuijk SJE. Active elimination of intestinal cells drives oncogenic growth in organoids. *bioRxiv* 2020; : 2020.11.14.378588.

29 Flanagan DJ, Pentinmikko N, Luopajärvi K, Willis NJ, Gilroy K, Raven AP *et al.* NOTUM from Apc-mutant cells biases clonal competition to initiate cancer. *Nature* 2021. doi:10.1038/s41586-021-03525-z.
